# Schools for Recent Graduates

**Published:** 1907-09-07

**Authors:** 


### SCHOOLS FOR RECENT GRADUATES.

### Opportunities in Special Hospitals.

### The London Post-graduate Association.

The "London Post-Graduate Association" issues tickets which admit to the clinical practice of a number of the leading general and special hospitals in the metropolis. The fee for a term of three months is £10 10s. Applications should be addressed to the Secretary, London Post-Graduate Association, Examination Hall, Victoria Embankment, W.C.

### Ophthalmic Hospitals.

Ophthalmic hospitals are the Royal London Ophthalmic Hospital, City Road, E.C.; the Royal Eye Hospital, St. George's Circus, S.E.; and the Royal Westminster Ophthalmic Hospital, King William Street, W.C. In each of these the fees charged are very modest, and full particulars can be obtained on application to the hospital secretary.

A word must be said of the holiday course in ophthalmology which is held at Oxford, in July. It extends over a fortnight, and the greater part of the day is occupied in practical work. Application should be made to Mr. R. W. Doyne, University Reader in Ophthalmology.

### North-East London Post-Graduate College.

This school, which is in connection with the Prince of Wales's General Hospital, Tottenham, N., is recognised by the University of London and the India Office as a place of post-graduate study. Opportunities are here afforded for attending demonstrations of various branches of medicine, surgery, and gynæcology, with opportunities for clinical instruction in diseases of the eye, ear, throat, nose, skin, and in fevers, diseases of children, psychological medicine, anæsthetics, and dentistry. Special classes with a limited attendance have been arranged for these subjects. The fee for a three months' course in any single department is one guinea, or three guineas admits for a similar term to the whole practice of the hospital. A perpetual ticket costs five guineas. Additional information, with syllabus of lectures and special classes, from the Dean, Dr. A. J. Whiting, 142 Harley Street, W.

### Other Hospitals.

University College has arrangements by which Dr. Vaughan Harley holds classes in practical pathological chemistry for the advantage of qualified practitioners and advanced students. Each student not only does the ordinary class analysis, but learns to apply the results to practice. The course includes analysis of saliva, stomach contents, fæces, urine calculi, and pathological fluids. The laboratory is open from 9 a.m. to 5 p.m. The fee for the class, including apparatus and material, is £5 5s. Charing Cross Hospital arranges three special courses of lectures and practical demonstrations during the year, each course consisting of ten meetings and lasting ten weeks. The class meets each Thursday at 4 p.m. in the board room of the Hospital, and thence proceeds to the department in which the demonstration may be held. Apply to Dr. Bosanquet at the Hospital.

### The Liverpool School of Tropical Medicine.

This school is carried on in conjunction with the University of Liverpool and the Royal Southern Hospital, for the purposes of research and clinical teaching respectively. A special ward at the hospital has been set apart for the reception and treatment of tropical diseases, and facilities for investigation and study are given in the Johnston Laboratories of the University. The fee for a full course (lasting about two months) is 10 guineas. A hall of residence is attached to the school.

### Queen Charlotte's Lying-in Hospital, Marylebone Road, N.W.

Qualified Medical Practitioners attending this hospital are permitted to do so for four weeks for a fee of £8 8s. The residence is at No. 2 Cosway Street, N.W. Terms for board and residence £1 15s. weekly.

### The Hospital for Diseases of the Skin, Blackfriars,

Gives practical courses of cutaneous histopathology and diagnosis and treatment of diseases of the skin and syphilis at frequent intervals. They are conducted by T. J. P. Hartigan, F.R.C.S. (Eng.). Apply, G. A. Richardson, Secretary.

### National Hospital for the Paralysed and Epileptic, Queen Square, W.C.

This hospital is a School of Medicine of the University of London, and has been recognised by the conjoint Board for England as a place where part of the fifth year may be devoted to clinical work. The out-patient practice is open without fee from 2.30 every day (except Thursday and Saturday), when patients are seen by the physicians for out-patients and the assist physicians. The in-patient practice is only open to those who take out the hospital course. Three courses of lectures and clinical demonstrations will be given in each year. The out-patient hospital practice and lectures are free, but for the hospital course a fee of two guineas for three months or three guineas for six months is charged. The museum is available for the prosecution of special work under the pathologist, for which a separate fee of two guineas for three months is charged. Apply C. E. Beevor, Dean of the Medical School.

### The Hospital for Women, Soho.

The hospital contains 60 beds. In the out-patient department there were over 4,000 new cases during the past year, the total number of out-patient attendances being 16,338. This large number affords opportunities for examining and studying most of the varieties of diseases peculiar to women. Clinical assistants receive notice of all operations performed within the hospital, and every facility is afforded them in the out-patient department of obtaining experience in diagnosis and treatment and the practical use of instruments. Fee for one month, three guineas, for each subsequent month, two guineas and a half. A certificate is given at the end of a three-months' course. Any further information can be obtained by writing to the Dean at the hospital.

Central London Throat and Ear Hospital, Gray's Inn Road, W.C.

Last year 354 in-patients were treated and 10,050 out-patients were seen, involving over 50,000 separate attendances. Operation days: In-patients and out-patients, Tuesday and Friday, at 2.30 p.m. Special courses of practical demonstrations are given weekly on Wednesdays during the winter and summer sessions by the members of the staff. They are so arranged that practitioners joining at any time are enabled to complete the group of subjects in a course of six weeks. The fee for this course is one guinea, or, with daily attendance at the out-patient department, two guineas. The fee for general clinical attendance is three guineas for three months and five guineas for six months. All information relating to teaching arrangements can be obtained on application to the Dean, Dr. Wyatt Wingrave. Apply to Richard Kershaw, Secretary.

Hospital for Diseases of the Throat, Golden Square, London, W.

Clinical instruction in the diagnosis and treatment of disease is given daily in the out-patient department from 2.30 to 5 p.m., on Tuesdays and Fridays from 6.30 to 9 p.m., and on Mondays at 9.30 a.m. Practitioners and medical students are admitted to the practice of the hospital at a fee of five guineas for three months, seven guineas for six months, or ten guineas for perpetual studentship. Apply to Charles A. Parker, Dean.

### IN SCOTLAND.

Post-Graduate Teaching in Edinburgh.

The four Scottish Universities, at three of which are situated medical schools of no mean reputation, have been but little distinguished in the past for their encouragement of post-graduate study.

St. Andrew's and Aberdeen concern themselves with the graduate no further than by offering him a degree in Public Health. Beyond this, Glasgow, offers courses in practical bacteriology, pathological histology, physiological chemistry, embryology, and a few of the special departments. Special courses are provided at Anderson's College Medical School and at St. Mungo's College. At the Glasgow Royal Infirmary the members of the staff of the hospital, with the hearty co-operation of the managers, have now arranged a sixth series of post-graduate classes. These are to be opened on September 3 by Sir Almroth E. Wright, M.D., F.R.S. The subject of his address is the "Principles of Vaccine-Therapy," one to which he has devoted much attention and original research. This year the staff of the Royal Infirmary have organised a most comprehensive series of practical and clinical classes. The previous courses have already proved very successful, and have been well attended. This present course will include in all about one hundred and twenty meetings. Apply to Dr. Maxtone Thom, Superintendent of the Royal Infirmary.

The Edinburgh Scheme.

In Edinburgh, however, a more elaborate scheme is available for the graduate desirous of continuing his studies. The University of this city, in addition to teaching the subject of public health, has for some years provided advanced classes in the ordinary medical subjects for senior students and graduates, such as bacteriology, organic chemistry, and embryology, while numerous laboratories attached to the various professorial chairs have been open for research under certain restrictions. These laboratories are increasing yearly in equipment and efficiency, mainly as a result of the funds derived from Mr. Andrew Carnegie's gift of £2,000,000 to the Scottish Universities. For this purpose, too, a magnificent laboratory has been organised and equipped by the Royal College of Physicians of Edinburgh, where any medical graduate may obtain permission to conduct scientific research.

The University and the Royal Colleges of Edinburgh provide courses for graduates who have recently obtained their diploma—medical officers home on furlough, practitioners resident in country districts, foreign doctors anxious to study English, and any others who may desire a brief *resumé* of the essentials and recent advances in various special subjects. The holiday course lasts from September 17 to October 6, and embraces lectures and demonstrations on some twenty or thirty subjects. The course has been arranged so that each member may attend a large number of subjects, for each of which a teacher has been chosen who is regarded as to some extent an expert in his subject. Most of the subjects are covered in three or six demonstrations of an hour or two hours each, and the fee charged is a composition one of six guineas for the whole course, or three guineas for any three subjects selected. Those attending the course may, if they so desire, obtain rooms in one of the University Halls at a charge of £1 10s. per week for board and lodging.

### IN IRELAND.

Trinity College, Dublin.

A three weeks' post-graduates' course is given during the summer session at Trinity College, Dublin. The fee is £5 5s. A limited number of the class can live in the college rooms and dine in hall, at a charge of £1 per week. Apply Dr. Alfred Parsons, Lower Fitzwilliam Street, Dublin. A school has been formed to prepare candidates for the Royal Navy, the Royal Army Medical Corps, and the Indian Medical Services. The classes are held twice a year. Apply Dr. C. A. K. Ball, Merrion Square, Dublin. An excellent post-graduate course is that of the Catholic University School of Medicine, given at the Mater Misericordiæ and St. Vincent's Hospitals, Dublin. The summer course commences on June 10, and the autumn course on September 15. The fee for the entire course is five guineas. Full particulars may be obtained from the Registrar, Medical School, Cecilia Street, Dublin.

Belfast.

Special summer classes are formed in bacteriology, clinical pathology, neurology, chemical physiology; and during the winter facilities are afforded to graduates to work at these subjects.

